# CRISPR/Cas9-Mediated Knockout of the *Dicer* and *Ago2* Genes in BHK-21 Cell Promoted Seneca Virus A Replication and Enhanced Autophagy

**DOI:** 10.3389/fcimb.2022.865744

**Published:** 2022-04-27

**Authors:** Xiaoyan Wu, Shuo Wang, Chen Li, Jianli Shi, Zhe Peng, Chang Liu, Hong Han, Yingru Ma, Limei Zheng, Shaojian Xu, Wei Du, Jun Li, Fan Zhang

**Affiliations:** ^1^Key Laboratory of Animal Resistance Biology of Shandong Province, College of Life Science, Shandong Normal University, Jinan, China; ^2^Division of Swine Diseases, Shandong Provincial Key Laboratory of Animal Disease Control & Breeding, Institute of Animal Science and Veterinary Medicine Shandong Academy of Agricultural Sciences, Jinan, China; ^3^Shandong Provincial Key Laboratory of Animal Cells and Developmental Biology, School of Life Science, Shandong University, Qingdao, China; ^4^College of Veterinary Medicine, Qingdao Agricultural University, Qingdao, China

**Keywords:** CRISPR/Cas9, Ago2, dicer, Senecavirus A, viral replication, autophagy

## Abstract

RNA interference (RNAi) is a major form of antiviral defense in host cells, and Ago2 and Dicer are the major proteins of RNAi. The Senecavirus A (SVA) is a reemerging virus, resulting in vesicular lesions in sows and a sharp decline in neonatal piglet production. In this study, CRISPR/Cas9 technology was used to knock out Ago2 and Dicer genes in BHK-21 cell lines used for SVA vaccine production. Cell clones with homozygous frameshift mutations of Ago2 and Dicer genes were successfully identified. The two knockout cell lines were named BHK-Dicer^Δ-^ and BHK-Ago2^Δ-^. Results showed that the two genes’ knockout cell lines were capable of stable passage and the cell growth rate did not change significantly. The replication rate and virus titers of SVA were significantly increased in knockout cell lines, indicating that RNAi could inhibit SVA replication. In addition, compared with normal cells, autophagy was significantly enhanced after SVA-infected knockout cell lines, while there was no significant difference in autophagy between the knockout and normal cell lines without SVA. The results confirmed that SVA could enhance the autophagy in knockout cells and promote viral replication. The two knockout cell lines can obtain viruses with high viral titers and have good application prospects in the production of SVA vaccine. At the same time, the RNAi knockout cell lines provide convenience for further studies on RNAi and SVA resistance to RNAi, and it lays a foundation for further study of SVA infection characteristics and screening of new therapeutic drugs and drug targets.

## Introduction

The ability of organisms to react to and resist foreign substances is essential for survival. Hosts have evolved many strategies of antiviral defense to inhibit viral replication. The discovery of RNA interference (RNAi) has been a major milestone with the form of sequence-specific gene silencing induced by double-stranded RNA (dsRNA) (Fire et al., 1998). RNA viruses, except retroviruses infection, lead to the generation of long dsRNAs during the virus life cycle (Umbach and Cullen, 2009). In the course of antiviral RNAi, dsRNA produced by viral replication is either a replication intermediate of viral dsRNA or a replication intermediate of viral RNA secondary structure cleaved by host endoribonuccinase (Dicer) enzyme into 21-23 nucleotides siRNA. These siRNAs, together with Ago2 protein and Dicer enzyme, form the induced silencing complex (RISC), which cleaves mRNA at the binding site. The cleaved mRNAs are then degraded and the host cells are induced to degrade these mRNAs. RNAi has been widely recognized as one of the most important antiviral mechanisms in fungi, plants, and invertebrates. In mammalian cells, viral dsRNAs and other viral nuclei acids primarily induce innate antiviral immune defenses, such as the interferon (IFN) system. Whether RNAi plays an important antiviral role in this process has long been a topic of debate (Cullen et al., 2013; Maillard et al., 2019).

The Senecavirus A (SVA), formerly named Seneca Valley Virus (SVV), was initially identified in the United States (US) in 2002 from a cell culture contaminant and belongs to the genus *Senecavirus*, family *Picornaviridae* (Hales et al., 2008). In 2016, the International Commission on Viral Taxonomy (ICTV) renamed it Senecavirus A. Historically, the cases of senecavirus-associated vesicular disease were reported in pigs in Manitoba, Canada in 2007 and a boar in the USA in 2012 (Pasma et al., 2008; Singh et al., 2012). Presently, SVA has been detected in pigs of different ages in Canada, Brazil, China, Colombia, Thailand, and other countries (Wang et al., 2017; Saeng-Chuto et al., 2018; Zhang et al., 2018; Arzt et al., 2019; Wang et al., 2019; Jiang et al., 2021), indicating that this disease has become a worldwide problem.

The evolution of RNAi across organisms is conserved, but its role in antiviral immunity varies from insect to mammal. Recent studies have shown that RNAi plays an important antiviral role in mouse embryonic stem cells and human neural progenitor cells (Maillard et al., 2013; Xu et al., 2019). Whether RNAi can play a role in SVA infection has not been reported.

Autophagy is a conserved catabolic process that is critical to maintaining the stability of the intracellular environment and has been identified as a major contributor to removing misfolded and aggregated proteins in the cytoplasm of mammalian cells (Rubinsztein, 2006; Mizushima et al., 2008; Kirkin et al., 2009). In recent years, studies have shown that autophagy plays an important role in antiviral immunity by targeting viruses and initiating host immune responses. Members of the picornavirus, including foot-and-mouth disease virus (FMDV) (Sun et al., 2018), cerebral myocarditis virus (EMCV) (Zhang et al., 2011; Hou et al., 2014), enterovirus 71 (EV71) (Tung et al., 2010), Coxsackie virus (CVB3), poliovirus (PV) (Velazquez et al., 2018; Mohamud et al., 2020), and SVA (Wen et al., 2021; Dong et al., 2021; Song et al., 2022) could promote viral replication by autophagy mechanism.

Depletion of Ago2 or Dicer led to a reduction of miRNA expression, and selective autophagy degraded Dicer and Ago2 and regulates miRNA activity (Gibbings et al., 2012), but impacts on loss of Dicer and Ago2 on autophagy was unknown.

In this study, CRISPR/Cas9 was used to successfully knock out the Ago2 and Dicer genes of BHK-21 cells. The replication rate of SVA in these two knockout cells were accelerated and the virus titer was significantly increased. The results were the first to demonstrate that RNAi was resistant to SVA replication in BHK-21 cells. In addition, the autophagy of the knockout cell lines without Dicer and Ago2 genes increased after SVA infection, while there was no significant difference in autophagy between the knockout and normal cell lines without SVA. The results suggested that SVA could enhance autophagy in knockout cells and promote viral replication.

## Materials and Methods

### Cell Lines, Virus, and Antibodies

BHK-21 cells (maintained in our laboratory) were cultured at 37°C 5% CO_2_ in DMEM basic (1×) (Gibco) supplemented with 10% fetal bovine serum (Biological Industries, Israel). The SVA strain (SVA-CH-SDGT-2017) and an anti-SVA-VP2 monoclonal antibody were prepared in our laboratory. The following antibodies were used: Ago2 (Abcam, ab186733), LC3B (Sigma-Aldrich, L7543), SQSTM1/p62 (Abcam, ab56416), goat Anti-rabbit IgG/HRP antibody (Solarbio, SE134).

### Generation of Ago2- and Dicer-Mutated BHK-21 Cells by the CRISPR/Cas9 System

The PCR primers used to construct single guide RNA (sgRNA) vectors are listed in **Table 1**. The gRNAs were inserted into pX458 vector by BbSI (NEB) digestion BHK-21 cells (5 × 10^5^ cells) in 6-well plates were transfected with each of the vectors (3.75µg) and the helper Lipo3000 (7.0µl) used. Forty-eight hours after plasmid transfection, the cell genome was extracted, and the target band was amplified by PCR with corresponding primers (**Table 2**). The PCR products were sent to Biosune Biotechnology (Shanghai) Co. Ltd. for sequencing and selecting the sgRNAs with the best shearing effect. The most suitable sgRNA was re-transfected into BHK-21 cells according to the previous conditions and 48 hours later, cells were digested, and the positive cells were sorted by flow cytometer (BD). The positive cells were cultured in a nutrient solution containing 1% penicillomycin in a 5% CO_2_ incubator at 37°C. The cells were counted during passage. The positive cells were divided into 96-well plates by limited dilution method. The genome of the monoclonal cell line was extracted with kit (Bioer) and amplified by PCR. PCR products were sent to be sequenced to identify homozygous deficient cells.

**Table 1:** sgRNA sequences.

name	sequence5’-3’	location
Dicer-1F	CACCGTGCTTTGCAGCCCCTCAGCA	Exon4
Dicer-1R	AAACTGCTGAGGGGCTGCAAAGCAC	
Dicer-2F	CACCGACCCCTGCTTCCTCACCAAT	Exon4
Dicer-2R	AAACATTGGTGAGGAAGCAGGGGTC	
Dicer-3F	CACCGGTCTGCTTGAACACCGGCTC	Exon5
Dicer-3R	AAACGAGCCGGTGTTCAAGCAGACC	
Dicer-4F	CACCGACCTCAACCCACGTGCAAAA	Exon5
Dicer-4R	AAACTTTTGCACGTGGGTTGAGGTC	
Ago2-1F	CACCGGTGCCGAAGTCCGGCCGAGG	Exon1
Ago2-1R	AAACCCTCGGCCGGACTTCGGCACC	
Ago2-2F	CACCGGCTTCCGGTCCCCGAAGATC	Exon2
Ago2-2R	AAACGATCTTCGGGGACCGGAAGCC	
Ago2-3F	CACCGCTTGTCCCTGCCGATTGGAA	Exon3
Ago2-3R	AAACTTCCAATCGGCAGGGACAAGC	
Ago2-4F	CACCGTGAAGGCTGCTCCAACCCCC	Exon4
Ago2-4R	AAACGGGGGTTGGAGCAGCCTTCAC	

**Table 2:** Amplified primer sequences.

name	sequence5’-3’	length
Dicer-sg1/sg2-F	GATTGCCTGGTTGAGTATAGATTGC	548bp
Dicer-sg1/sg2-R	GCAGACGTTAGAACAGAAAAGAACA	
Dicer-sg3/sg4-F	AGCCATTTTACTCCCCTGTTATTCT	723bp
Dicer-sg3/sg4-R	TCAGACACTAAGCTTCCTCAGTAAC	
Ago2-sg1-F	AGAACATGGAGAAGATATTGGTGGG	544bp
Ago2-sg1-R	TACATCACAGCAGTTTCATGGTAGA	
Ago2-sg2/sg3-F	CTCTGTCTCAGGGAAATAGTGGAG	723bp
Ago2-sg2/sg3-R	ACATAGTACGTTCCTGTCAGTATGG	
Ago2-sg4-F	GTGTGGAACAGTGAGGCTGA	739bp
Ago2-sg4-R	GTGGCCCCAACCCTAATACC	

### Cell Proliferation Assay

BHK-21, BHK-Dicer^Δ-^and BHK-Ago2^Δ-^ were cultured in a 96-well plate at 37°C with a concentration of 1 × 10^4^ cells/well. At 12, 24, and 36 hours after incubation, 10µl of solution in cell counting kit-8 (CCK-8, MEILUNBIO) was added to each well. The cells were incubated at 37°C for 2 h. Details about the experimental procedures were described in the manual of CCK-8. The absorbance at 450nm was measured by a microplate reader (Bio-Red). Data are representative of three independent experiments performed in triplicate.

### Western Blot Assay

Harvested cells were washed with PBS and lysed with Cell lysis buffer for Western and IP (Beyotime, P0013) containing 1 mM phenylmethanesulfonyl fluoride (PMSF) (Beyotime, ST506-2) and centrifuged. Twenty micrograms of protein were separated using SDS-PAGE and transferred to nitrocellulose membranes (Pall, 66485), and then blocked with NcmBlot blocking buffer (NCM Biotech, P30500) for 10 min at room temperature (RT). The membranes were subsequently reacted with primary antibodies at 4°C for 12 h, and then incubated with horseradish peroxidase-conjugated secondary antibodies for 1 h at RT. Proteins on the membranes were detected using a SuperSignal West Pico PLUS Chemiluminescent Substrate Kit (ZETA) and visualized *via* a chemiluminescence apparatus (ImageQuant LAS500.GE).

### Effect of Knockout Cells on SVA Growth Curve, TCID_50_ Assay, and Autophagy

To determine viral replication kinetics in knockout cell lines, virus growth experiments in BHK-21, BHK-Dicer^Δ-^and BHK-Ago2^Δ-^ cells were done. The cell monolayers in 6-well tissue culture plates were washed with PBS and infected with SVA virus (0.01 MOI; Equal to PFU number/cell). The infected cells were incubated at 37°C and harvested at different times. The plates were subjected to three successive freeze-thaw cycles to remove cell fragments by centrifugation. Tenfold serial dilutions of viruses harvested at different times were prepared in DMEM. Fifty percent tissue culture infective dose (TCID_50_) values were determined by the Reed-Muench formula (Reed and Muench, 2018). Mean values and standard deviations were calculated from the results of three independent experiments. RNA of the virus was harvested at different time points. The amount of virus was determined by SYBR Green qPCR using the follow primers.

(SVA-QRTU: 5’-AGAATTTGGAAGCCATGCTCT-3’;
SVA-QRTL: 5’-GAGCCAACATAGAAACAGATTGC-3’)

Protein samples collected at different time points were detected according to Western blot assay.

## Results

### Using CRISPR/Cas9 Expression Vector to Knock Out Dicer and Ago2 Genes in BHK-21 Cells

**Figure 1:**
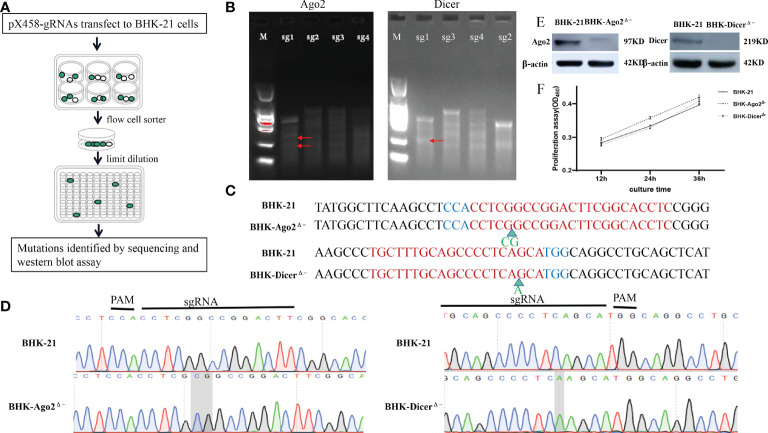
Construction and identification of knockout cell lines. **(A)** Schematic of the construction process of knockout cell lines. After transfection with the plasmids targeting Dicer and Ago2 protein genes into BHK-21 cells, cell sorting was performed by flow cytometry. After 1-2 days, stable cell lines containing various mutant cells and normal cells were obtained, with limiting dilutions by 96-well plates several times (diluted as 10 cells/well every time), mutant cells were enriched. Mutations were confirmed by sequence analysis and Western blot assays. **(B)** T7EI assay was performed to detect the mutants. White arrows indicate the predicted sizes of T7E1 digestion. Primers used or the PCR reactions are listed in **Table 2**. The bands marked by the red arrows are the bands cut by the T7E1 enzyme. **(C)** Partial sequence alignment results of mutant Ago2 and Dicer near mutant sites in BHK-21 and knockout cell lines. Red color-marked nucleic acids indicate gRNA target sequences, while the blue ones indicate PAMs. Green arrows represent the inserted bases. **(D)** Sequencing peak diagram of mutant Ago2 and Dicer near mutation sites in BHK-21 cells and knockout cell lines. **(E)** Identification of Ago2 and Dicer protein. The Ago2 and Dicer protein could not be detected in the knockout cell lines, proving that Ago2 and Dicer genes were completely knocked out. **(F)** Knockout of Ago2 and Dicer genes did not significantly affect the cells’ proliferation. The numbers in the y-axis represent fold changes derived from the OD values by measuring the absorbance at 450nm using a microplate reader. Data are expressed as the mean ± SEM from three independent experiments.

To construct Ago2 and Dicer gene knockout cells, using http://crispor.tefor.net/, sgRNAs targeting the Dicer gene (Gene ID:101839371) and Ago2 gene (Gene ID: 101843722) were designed. Due to the U6 promoter for gRNA expression, all gRNAs were added CACCG at 5’, the reverse complementary sequence was added AAAC at 5’ and a C base at 3’. The upstream and downstream sgRNAs primers were paired by the stepwise annealing method (**Table 1**). T7E1 enzyme digestion to determine the sgRNA with the best shearing effect was shown in **Figure 1B**. After pX458-gRNAs transfect into BHK-21 cells, due to eGFP carried on pX458 vector, cell sorting and limited dilution were performed, as shown in **Figure 1A**. Stable homozygous deletion cell lines were obtained by sequenced (**Figure 1C**). Ago2 had a 2-base insertion at the pre-cut position of Cas9 (between the third and fourth bases of PAM), whereas Dicer had a 1-base insertion at the pre-cut position of PAM (between the fifth and sixth bases of PAM) (**Figures 1C, D**). Such frameshift mutations are expected to result in a frameshift, premature termination, and loss of function of protein translation. The quality of the peak maps near the mutation sites is very high (**Figure 1D**). The Ago2 and Dicer protein were successfully knocked out (**Figure 1E**).

We also investigated cell growth with a cell counting kit (CCK-8). As shown in **Figure 1F**, compared with BHK-21 cells, there was no significant change in the proliferation rate of knockout cells.

### The SVA Does Not Exhibit Reduced Fitness in Knockout Cell Culture

**Figure 2:**
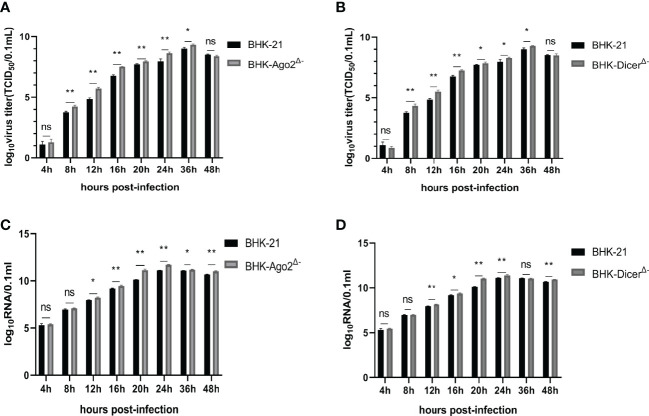
Knockout cells promote the ability of SVA replication. **(A, C)** Replication ability of SVA in BHK-21 cells and BHK-Ago2^Δ-^ cells. The cells were infected with SVA with the SVA at MOI of 0.01. The viruses harvested at different times were titrated and expressed as TCID_50_, and the genome copy numbers were measured for the same samples by real-time reverse transcription-PCR (RT-PCR). Mean ± SD values are shown (repeated-measures ANOVA, n = 3; ns, no significant difference; *P < 0.05; **P < 0.01). **(B, D)** Replication ability of SVA in BHK-21 cells and BHK-Dicer^Δ-^ cells. The cells were infected with SVA with the SVA at MOI of 0.01. The viruses harvested at different times were titrated and expressed as TCID_50_, and the genome copy numbers were measured for the same samples by real-time reverse transcription-PCR (RT-PCR). Mean ± SD values are shown (repeated-measures ANOVA, n = 3; ns, no significant difference; *P < 0.05; **P < 0.01).

The growth characteristics of SVA during a single replication cycle in BHK-21, BHK-Dicer^Δ-^ and BHK-Ago2^Δ-^ cells were examined. In BHK-Dicer^Δ-^ and BHK-Ago2^Δ-^ cells, there were significant differences in the yield of infected than in BHK-21 (**Figures 2A, B**). In addition to the production of infected viruses, we also quantified total genomic RNA by real-time PCR. The viruses produced higher levels of RNA in both knockout cell types (**Figures 2C, D**). The results showed that the replication rates and virus titers of SVA were significantly increased in knockout cell lines.

### Autophagy Intensified After SVA Inoculation of Knockout Cell line

**Figure 3:**
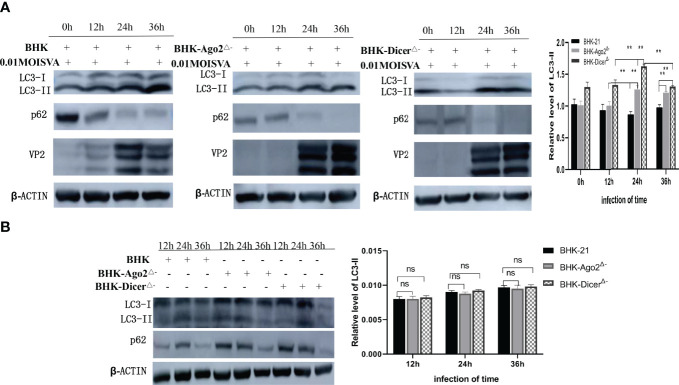
Autophagy promotes the proliferation of SVA in knockout cells. **(A)** Western blot analysis of LC3, p62, VP2, and β-actin levels in transfected BHK-21 cells and knockout cells that were mock-infected with SVA at an MOI = 0.01. The relatative level of LC3-II from three independent experiments were shown (ns, no significant difference; **, P < 0.01). ImageJ was used to quantify the level of protein. **(B)** Western blot analysis of LC3, p62, and β-actin levels in transfected BHK-21 cells and knockout cells. The relative level of LC3-II from three independent experiments were shown (ns, no significant difference). ImageJ was used to quantify the level of protein.

The conversion level of LC3-I to LC3-II was significantly upregulated in SVA-infected cells compared with mock cells (Velazquez et al., 2018; Mohamud et al., 2020). To investigate whether SVA infection in knockout cell lines induces autophagy, we detected the expression level of LC3-I, LC3-II, and SQSTM1/p62 in BHK-Dicer^Δ-^ and BHK-Ago2^Δ-^ cells. It was found that the LC3-II transformation level was significantly up-regulated in knockout cells compared with normal BHK-21 cells after SVA infection (**Figure 3A**). The results showed that SVA infection in knockout cell lines could induce autophagy and enhance autophagy. In addition, VP2 protein expression represented the progression of SVA infection (**Figure 3A**). VP2 was detected 12 hours after we infected cells with 0.01MOI virus. As shown in **Figure 3A**, beginning at 12 hpi, the densitometric ratio of the β-actin and LC3-II bands, an accurate indicator of autophagic activity, was much higher in SVA-infected BHK-Dicer^Δ-^ and BHK-Ago2^Δ-^ cells than in the corresponding mock-infected BHK-21 cells, indicating that SVA infection promoted autophagy (*P* < 0.01). These results confirmed that SVA infection in BHK-Dicer^Δ-^ and BHK-Ago2^Δ-^ cells intensified autophagy.

## Discussion

CRISPR/Cas9 gene editing technology is an important tool for gene function research and suitable drug targets, while knocking out genes in cells may affect cell activity and proliferation. Previous studies have shown that knockout of the Dicer gene in EPC cells significantly reduced cell growth, proliferation, and activity (Kwak and Kim, 2021). In this study, CRISPR/Cas9 technology was used to knock out Ago2 and Dicer genes in BHK-21 cell lines used for SVA vaccine production. Cell clones with homozygous frameshift mutations of Ago2 and Dicer genes were successfully identified. It was proved that the deletion of Ago2 and Dicer genes did not affect cell proliferation (**Figure 1F**). We speculate that the difference was due to the different cell lines.

The ability of an organism to respond to and resist foreign substances is necessary for survival. Hosts have evolved a number of antiviral defense strategies to inhibit viral replication. RNAi is one of the innate defenses of host cells. In mammalian cells.

RNA virus infection induced a typical antiviral RNAi response, characterized by the production of viral siRNAs with clearly defined properties of canonical siRNAs (Li et al., 2013). In our study, the data showed that the replication rate and virus titers of SVA were significantly increased in knockout cell lines (**Figure 2**), suggesting that RNAi could inhibit viral replication. Some researchers have studied the relationship between viral proteins and RNAi. Yang Qiu and Yuan Fang et al. had confirmed that HEV71 3A protein was a true RNAi inhibitor in the process of viral infection (Qiu et al., 2018; Fang et al., 2021). When 3A-mediated RNAi inhibition was impaired or inhibited, viral replication was reduced. This effect disappeared in the absence of the Dicer or Ago2 gene. These findings had parallelized with the induction and suppression of antiviral RNAi by the Coxsackievirus (Fang et al., 2021). We hypothesized that SVA 3A protein also had a similar anti-RNAi effect.

Autophagy is a double membrane vesicle in degradation of damage and harmful ingredients, in order to maintain cell metabolism balance and steady (Yang and Klionsky, 2010; Klionsky and Codogno, 2013).

Although autophagy was initially thought to be nonselective, there is considerable evidence for selective autophagy degradation of cytoplasmic components (Johansen and Lamark, 2011).The studies have found that Dicer and Ago2 were degraded by selective autophagy receptor into miRNA-free entities (Gibbings et al., 2012). Autophagy established a checkpoint for continued miRNA loading into Ago2, and NDP52 and autophagy were required for the homeostasis and activity of the measured miRNA (Gibbings et al., 2012). Autophagy was also involved in the post-transcriptional regulation of Dicer mRNA. What is the relationship between RNAi and autophagy after cell infection with the virus? Recent studies have shown that SVA VP1, VP3, and 3C protein Akt-AMPK-MAPK-MTOR pathways played a synergistic role in inducing autophagy, thus promoting viral replication (Song et al., 2022). Wen et al. found that selective autophagy receptor SQSTM1/P62 inhibits SVA virus replication by targeting VP1 and VP3 (Wen et al., 2021). Moreover, SVA can use 2AB protein to inhibit autophagy, thus promoting virus replication in the later stage of SVA infection (Dong et al., 2021). In our studies, a normal cell line and two knockout cell lines were infected with SVA, VP2, LC3-I, LC3-II, and SQSTM1/p62 proteins were detected. Results showed that the expression of SVA VP2 was increased, and autophagy was enhanced in knockout cells (**Figure 3A**). At the same time, there was no significant difference in autophagy between normal and knockout cell lines (**Figure 3B**). Our findings further illustrate that SVA infection could induce autophagy and promote viral replication in knockout cells, and RNAi did not affect autophagy.

In conclusion, the interaction between host cells and foreign viruses was mutual. While the host adopted a variety of strategies to resist the invasion of foreign viruses, the viruses also found ways to respond to these strategies. This study first found that RNAi could inhibit SVA replication, and SVA infection RNAi free cell lines could induce autophagy and promote viral replication. In addition, RNAi did not affect autophagy. We speculate that SVA may act on RNAi through 3A protein, and the RNAi knockout cell lines constructed lay a foundation for further studies on RNAi and SVA resistance to RNAi.

## Data Availability Statement

The original contributions presented in the study are included in the article/supplementary material. Further inquiries can be directed to the corresponding authors.

## Author Contributions

JL and CheL conceived the project. XW designed the experiments. XW performed most of the experiments. SW, JS, ZP, ChaL, SX, WD, and HH contributed materials and participated in discussion. XW wrote the manuscript. JL and FZ supervised the work and edited the final version of the manuscript which was read and approved by all authors.

## Funding

The study was partly supported by Major Scientific and Technological Innovation Project (MSTIP) (2019JZZY010720), Shandong Province Pig Industry Technology System (SDAIT-08-06), Shandong Province agricultural applications of major innovation projects (SD2019XM003, SD2019XM006), Taishan Scholars Project, Agricultural Science and Technology Innovation Project of Shandong Academy of Agricultural Sciences (CXGC2018E10).

## Conflict of Interest

The authors declare that the research was conducted in the absence of any commercial or financial relationships that could be construed as a potential conflict of interest.

## Publisher’s Note

All claims expressed in this article are solely those of the authors and do not necessarily represent those of their affiliated organizations, or those of the publisher, the editors and the reviewers. Any product that may be evaluated in this article, or claim that may be made by its manufacturer, is not guaranteed or endorsed by the publisher.
